# Multimodal Management of Coexisting Atlantoaxial Subluxation and Spinal Stenosis in an Older Adult: A Case Report and Literature Review

**DOI:** 10.7759/cureus.51442

**Published:** 2024-01-01

**Authors:** Kristy Yau, Eric Chun-Pu Chu, Jason G Zhao, Linda Yin-King Lee, Cliff Tao

**Affiliations:** 1 Chiropractic and Physiotherapy Centre, New York Medical Group, Hong Kong, CHN; 2 School of Nursing and Health Studies, Hong Kong Metropolitan University, Kowloon, HKG; 3 Department of Radiology, Private Practice of Chiropractic Radiology, Irvine, USA

**Keywords:** anterolisthesis, chiropractic, spinal stenosis, atlantoaxial subluxation, c12 subluxation

## Abstract

This case report investigates the diagnostic and therapeutic challenges caused by the coexistence of atlantoaxial subluxation and spinal stenosis in a 70-year-old male patient with chronic progressive numbness in both hands. A detailed assessment showed that the patient's symptoms were primarily caused by spinal stenosis, not atlantoaxial subluxation. Considering the patient's age and preference for nonsurgical treatment, a conservative chiropractic care plan was implemented, significantly improving his symptoms and quality of life. This case highlights the potential benefit of conservative chiropractic care in managing such complex cases, emphasizes the need for meticulous diagnosis, and requires further research to validate these findings and develop comprehensive management guidelines.

## Introduction

The coexistence of atlantoaxial subluxation and spinal stenosis in the same patient, particularly in older patients, presents a unique diagnostic and therapeutic challenge. Atlantoaxial subluxation, marked by a loss of stable articulation and unprovoked excessive movement at the C1-C2 junction, can cause neck pain, headache, movement restriction, weakness, numbness, and pyramidal signs in many patients [1,2]. This condition commonly coexists with conditions such as rheumatoid arthritis, abnormalities in the upper cervical spinal structures, Down syndrome, and infection [1-3]. However, it is rare and difficult to diagnose when unassociated with traumatic, congenital, or rheumatic disorders [2].

In contrast, spinal stenosis, often associated with osteoarthritis of the spine, results from increased biomechanical loading of the anterior vertebral bodies and loss of normal lordotic position, causing abnormal structural alignment and motion [4,5]. The vertebral canal narrowing resulting in nerve compression can present with radicular symptoms such as pain, numbness, weakness, and cramping [6]. The overlapping symptoms of these conditions necessitate meticulous clinical examination, a high index of suspicion, and appropriate imaging for an accurate diagnosis.

Surgery is often the preferred treatment for atlantoaxial subluxation, particularly in patients with neurological compromise [1,2]. However, surgical decision-making is multifactorial and influenced by patient age, symptomatology, overall health, comorbidities, and preferences.

Considering these complexities, we present the case of a 70-year-old male patient diagnosed with atlantoaxial subluxation and spinal stenosis. This study aimed to shed light on the diagnostic difficulties encountered because of overlapping symptoms and management complexities when the standard surgical treatment is not the patient’s preferred option. Through this case report, we aimed to provide insights into the potential role of conservative chiropractic care in managing such complex cases while emphasizing the need for further research in this area.

## Case presentation

A 70-year-old male, a retired manager, presented to a chiropractic clinic with chronic progressive numbness in both hands over six months. This numbness, which was more pronounced on the right side, started three years ago and gradually involved the left side over the last six months. He described the symptoms as fluctuating in severity throughout the day but generally worsened in the morning. Numbness was particularly noticeable on the lateral aspects of both palms.

The patient reported difficulty performing fine motor tasks, such as buttoning a shirt, especially with the right hand. This symptom progressed concurrently with numbness. The patient did not report any associated pain or changes in the color of the hands or fingers. No lower limb symptoms were observed, indicating the symptoms were localized in the hands.

He had no known triggers or mitigating factors for these symptoms and denied any preceding trauma or injury to the hands or neck. He denied systemic symptoms, such as fever, weight loss, or fatigue, suggesting an underlying systemic disease.

The patient had been previously diagnosed with prostate cancer and treated with radiotherapy. His medical history was unremarkable, with no similar symptoms, allergies, or surgeries. Moreover, he was a nonsmoker and did not consume alcohol. He maintained a routine of simple stretching daily at home.

**Figure 1:**
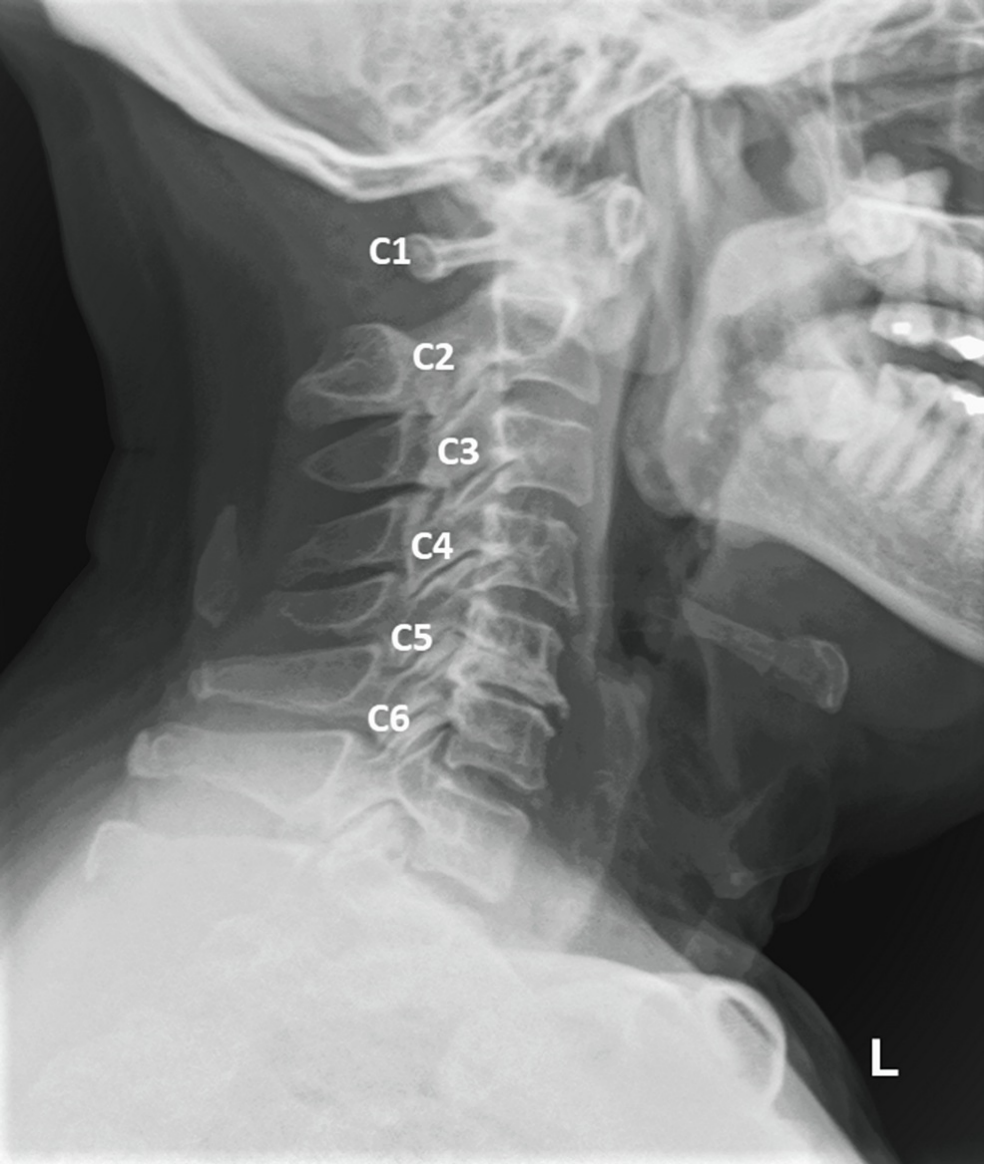
Cervical spine X-ray The cervical spine X-ray reveals multiple degenerative changes and alignment issues. Grade I anterolisthesis is evident at the C3 upon C4 level, indicating a forward slippage of the C3 vertebra over the C4 vertebra. Additionally, signs of cervical spondylosis are noticeable and characterized by anterior marginal osteophytes. The X-ray also demonstrates decreased disc height at the C5/6 and C7/T1 levels, suggesting an ongoing degeneration of intervertebral discs at these sites. Furthermore, an atlantoaxial (C1/2) subluxation is visible at 3.3 mm, which presents an abnormal alignment or movement between the atlas and the axis cervical vertebrae.

Six months before the presentation, the patient consulted an orthopedic surgeon for the same complaint. Cervical radiographs revealed atlantoaxial subluxation, grade I anterolisthesis at C3-C4, cervical spondylosis, and reduced disc height at C5/C6 and C7/T1. Surgery was suggested; however, the patient opted for conservative care following a chiropractic consultation (Figure 1).

During the initial chiropractic consultation, the neurological examination of the upper limbs revealed bilateral present and symmetric sensations, a reduced sensation in both hands, more pronounced on the right side. Motor strength was 5/5 bilaterally. Deep tendon reflexes were 1+ for the right biceps, 2+ for the left biceps, 1+ bilaterally for the brachioradialis, and 1+ bilaterally for the triceps. Hoffman’s sign was negative, and cranial nerve screening was unremarkable.

**Figure 2:**
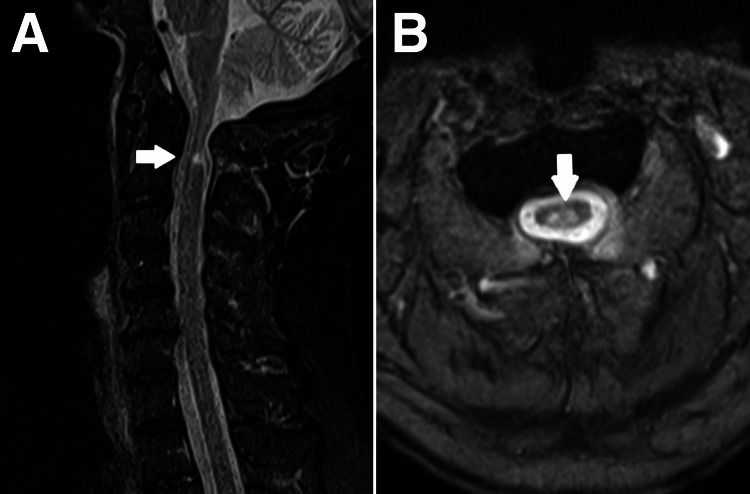
Cervical magnetic resonance image (MRI) Magnetic resonance image of cervical spine. Focal intramedullary T2W signal seen in the dorsal part of the spinal cord at C1 level suggesting cord edema or myelomalacia change. (A) At the C1 level, the atlantoaxial subluxation causes moderate central canal stenosis. (B) AP diameter of the thecal sac measures 6.9mm. An abnormal intramedullary T2W signal is seen at the dorsal part of the spinal cord at the C1 level, suggestive of cord edema or myelomalacia change.

**Figure 3:**
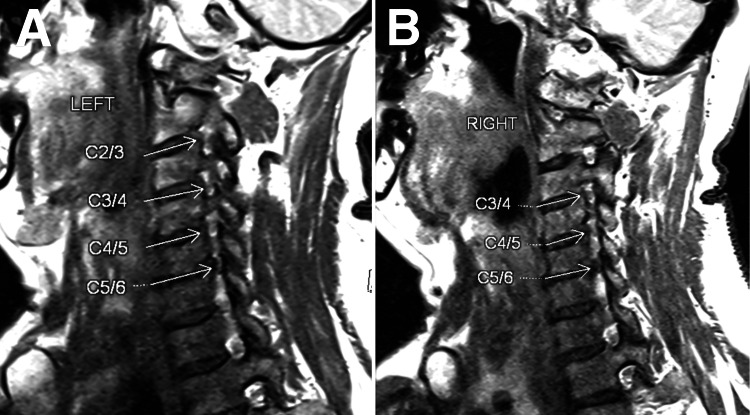
At C3/C4, C4/C5, and C5/C6 levels, a minimal posterior disc bulge indenting the thecal sac without causing significant spinal stenosis was noted. Bilateral foraminal stenosis was identified

A cervical magnetic resonance image (MRI) was performed to confirm atlantoaxial subluxation with moderate central canal stenosis at the C1 level (Figure 2) and bilateral foraminal stenosis at the C3/C4, C4/C5, and C5/C6 levels (Figure 3).

The patient was then started on a conservative management plan, including education; soft tissue therapy; lower cervical and upper thoracic joint low-velocity, low-amplitude mobilization; and a specific neck-strengthening exercise regimen. The specifics of the exercises and how they were performed at home were thoroughly explained to patients. High-velocity, low-amplitude manipulations of the cervical spine were not performed. The patient was advised to monitor neurological progression and return for reevaluation.

After six chiropractic treatments over two weeks, the patient reported a 50% improvement in numbness and difficulty in hand coordination. He continued with six additional chiropractic treatments for over one month, focusing on neck strengthening exercises and home exercises. Six weeks after the initial consultation, the patient reported 90% resolution of numbness and difficulty with hand coordination and reported no difficulty performing normal activities. He was discharged and advised to return if symptoms worsened.

**Figure 4:**
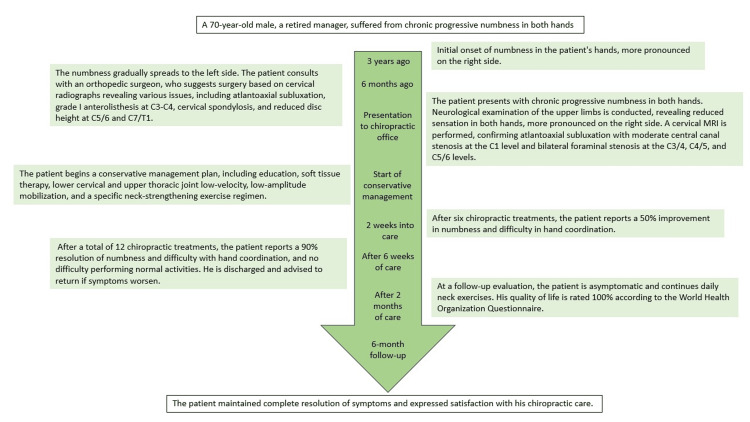
Timeline of care

At a follow-up evaluation two months later, the patient was asymptomatic and continued daily neck exercises. His quality of life, as measured by the World Health Organization Questionnaire, was rated 100%. The patient maintained complete resolution of symptoms at the six-month follow-up and expressed satisfaction with his chiropractic care (Figure 4). Plans for ongoing monitoring of the patient's condition were also initiated because of the chronic and progressive nature of his spinal conditions.

## Discussion

As described in this case, simultaneous manifestations of atlantoaxial subluxation and spinal stenosis can cause intricate diagnostic conundrums. Despite the common occurrence of radiographic atlantoaxial subluxation changes, the incidence of neurological injuries remains relatively rare [1]. Our patient's symptoms were predominantly attributed to spinal stenosis rather than a neurologically impactful atlantoaxial subluxation. This diagnostic distinction is vital in developing therapeutic strategies to address symptomatology. The trial treatment plan was tailored primarily for spinal stenosis, whereas atlantoaxial subluxation was meticulously monitored for potential severe complications.

A comprehensive review of the existing literature was conducted to identify cases involving patients with atlantoaxial subluxation who sought treatment from chiropractors. This review includes a thorough examination of case histories while omitting non-peer-reviewed sources. On August 20, 2023, a search was conducted across PubMed, Google Scholar, and Index to Chiropractic Literature databases from 2000 until August 2023 in the English language. Notably, various permutations of the following phrases were utilized: "chiropractor," "atlantoaxial subluxation," "atlantoaxial dislocation," and "atlantoaxial instability." We searched and found seven cases (Table 1) [2,7-12]. The total number of patients under consideration, including the present case, amounted to eight, with a mean age of 55±41; 75% of the patients identified as female. The predominant first manifestation was cervical discomfort, reported by 62% of the patients. Among the instances wherein the patient’s medical history was documented (n=7), two individuals had a history of rheumatoid arthritis.

**Table 1:** Cases involving patients with atlantoaxial subluxation who sought treatment from chiropractors

Author (year)	Sex	Age in years	Presentation	Neurological complication	Medical history	Treatment	Investigations	Radiological identification	Follow-up
Bonic 2010 [7]	F	67	Chronic neck pain and recent onset of dysphagia.	None	Rheumatoid arthritis	Surgical stabilization fusion	Computed tomography and magnetic resonance imaging	Significant bone destruction, gross joint derangement, and basilar invagination.	Positive after six months
Marchand 2014 [2]	F	83	Neck pain and headaches	None	High blood pressure and high cholesterol	Education, mobilizations, soft tissue therapy, monitoring for neurological progression, and co-management with the family physician	Cervical and open-mouth odontoid radiographs	Atlantodental space measuring 6 mm	Positive after 13 months
Chu 2018 [8]	M	47	Right hemicranial pain and tingling of the right face and ipsilateral hemitongue	None	None	A chiropractic adjustment, ultrasound therapy, and trigger point therapy	Cervical and open-mouth odontoid radiographs	Narrowing of the right paraodontoid space and degenerative changes in the lower cervical spine	Positive after three months
Chu 2019 [9]	F	70	Neck stiffness and swallowing problems	None	NA	Cervical mobilization and strengthening exercises	Cervical radiographs	Positive Ranawat index	Positive after six months
Chu 2020 [10]	F	14	Left head tilt and bilateral neck pain	None	None	Thermal ultrasound therapy, massage and muscle mobilization, light force cervical adjustment	Cervical and open-mouth odontoid radiographs	A tilt of the C2 median line, asymmetry of the C1 lateral masses, and of the atlantodens intervals	Positive after nine months
Chu 2020 [11]	M	10	Repetitive episodes of torticollis	None	None	Complete osteochondroma excision	Open-mouth odontoid radiograph and computed tomographic (CT)	Lateral displacement of the C2	Positive after 24 months
Chu, 2021 [12]	F	80	Head dropping and losing balance while walking	None	Rheumatoid arthritis	Gentle mobilization of the upper thoracic spine, electrical muscle stimulation (EMS) of the cervical extensors, and home-based neck exercises	Cervical radiographs	Anterior subluxation of the C1 on C2	Positive after four months
Current case	F	70	Bilateral hand numbness	Yes	None	Patient education, soft tissue therapy, lower cervical and upper thoracic joint low-velocity, low-amplitude mobilizations, and exercises	Cervical magnetic resonance imaging and radiographs	Atlantodental space measuring 3.3 mm, cord edema at C1	Positive after six months

In this case, effective management with conservative chiropractic care emphasizes its potential applicability when spinal stenosis is the predominant symptom. Chiropractic interventions, including patient education, soft tissue therapy, mobilization, and exercise, have substantially improved the patient's condition, highlighting the potential efficacy of nonsurgical approaches.

The outcomes of this case, in contrast with those in the existing literature, reveal considerable variability in treatment responses. Surgery is often the first-line intervention for atlantoaxial subluxation [1]. However, chiropractors frequently encounter and manage patients with similar spinal conditions [2,7-12]. This case underscores that conservative management strategies, like chiropractic care, can yield positive outcomes when symptoms are primarily driven by spinal stenosis.

This case contributes valuable insights to the literature and bolsters evidence for conservative chiropractic care in managing intricate spinal disorders. It emphasizes the importance of an accurate diagnosis to guide treatment decisions. Further research is required to confirm these findings and establish comprehensive management protocols for similar cases.

## Conclusions

This case highlights the diagnostic challenges posed by the coexistence of atlantoaxial subluxation and spinal stenosis, the latter being the primary source of the symptoms. Successful management of the patient's symptoms through conservative chiropractic care accentuates its potential role in cases where spinal stenosis is the primary cause, especially when surgical interventions may not be suitable or preferred. This case suggests the need for meticulous diagnosis in such complex scenarios to accurately identify the primary source of symptoms and for further research to validate these findings and develop comprehensive management guidelines. The results also emphasize the potential value of conservative treatment approaches, including chiropractic care, highlighting the importance of patient-centered care and personalized treatment plans. Future research, including longitudinal studies and randomized controlled trials, should explore the efficacy and safety of conservative chiropractic treatments for managing complex spinal conditions.
